# Biomechanically designed Curve Specific Corrective Exercise for Adolescent Idiopathic Scoliosis gives significant outcomes in an Adult: A case report

**DOI:** 10.3389/fresc.2023.1127222

**Published:** 2023-03-30

**Authors:** Sujata Maharathi, Raju Iyengar, Patnala Chandrasekhar

**Affiliations:** ^1^ Deptartment of Orthopaedics, Nizam’s Institute of Medical Sciences, Hyderabad, India; ^2^ Swami Vivekananda National Institute of Rehabilitation Training and Research, Olatpur, Cuttack, Odisha, India

**Keywords:** biomechanically designed curve specific corrective exercise for Adolescent Idiopathic Scoliosis (AIS), spinal pain syndrome of scoliosis, pelvic obliquity, rib hump, breathing with core, local and global muscles, global spinal balance, ergonomics for scoliosis

## Abstract

**Background:**

This study presents findings on improvements to the Frontal and Sagittal Cobb angle, Global Spinal balance, and lung function parameters (FEV1, PEF) in an adult male with idiopathic scoliosis suffering from pain during ADL and sports activities who was treated with a biomechanically designed exercise protocol.

**Case Presentation:**

The 26-year-old male reported upper and middle back pain which worsened when playing cricket. Whole spine standing x-Ray AP view revealed a right thoracic Scoliosis (Lenke 1 curve) of Cobb angle 48.6° with left lumbar compensatory curve of 24.7°, Thoracic hypo kyphosis of 9.9°, and VAS rating for pain of 8. The patient was treated with myofascial release, stretching, aerobics, strengthening exercises, “Breathing with core” for stabilization, and biomechanically designed curve specific corrective exercises.

**Results:**

Re-assessment 32 weeks post intervention demonstrated significant reduction in the major Cobb angle by 13.8°, minor Cobb angle by 9.5°, Thoracic hypokyphosis normalized to 37.8°, Coronal balance improved by 17.4 mm, Sagittal balance regained by 4.2 mm, Spine ROM improved by a total of 6.5 cm, Enhancement of pulmonary function of FEV1 by 7% and PEF by 18 litres/min, and dramatic improvement in aesthetics and pain perception.

**Conclusion:**

The biomechanically designed exercise protocol helped straighten the curve through curve specific corrective exercises and stabilized the curve by “Breathing with core”. It also treated the associated signs and symptoms of spinal pain syndrome by myofascial release and proper ergonomics, pulmonary dysfunction by aerobics, and muscle tightness and weakness (due to altered length-tension) by stretching and strengthening.

## Introduction

Scoliosis is a three-plane deformity of the spine, rib cage, and pelvis. Diagnosis is confirmed when the Cobb angle is 10° or more with recognizable vertebral axial rotations. After elimination of the other 20% known causes of scoliosis, when overall assessment and investigations fail to provide a definite reason for the development of the curve, it is diagnosed as Idiopathic Scoliosis. Among the three types, Adolescent Idiopathic Scoliosis is the most common and manifests and progresses during growth spurts in adolescence.

A major percentage of patients diagnosed with Adolescent Idiopathic Scoliosis require conservative management, with the primary goal of restricting curve progression during growth and also reducing the curve. But many adult cases remain untreated due to lack of diagnosis in their adolescence and so need to be treated during adulthood. Curve progression during adulthood is documented and understood as an asymmetrical spine alters the biomechanical loading, causing the viscoelastic structures to undergo creep which initiates the structural bony changes and muscular deterioration. Therefore, bigger curves >30° are the victims of curve progression (1) as the creep effect increases proportionately with curve size.

So, exercises are in great need not only during growth and development but also during maturity and adulthood. A sound knowledge of spine biomechanics and the associated deformities is essential for comprehending and formulating exercise methods. It is also imperative to understand curve types, as the exercises are curve-specific.

Among all classification systems, Lenke classification (2) is the most comprehensive and popular among orthopedic surgeons, and hence is used for this case. The bending /traction radiographs that are used in the Lenke system to detect the structural component of a curve (if it is >25°) are useful for conservative treatment as they give prognostic information regarding the possibility of curve correction.

The difference between lying and traction view reveals the degree of correctability of the concavity side soft tissues and the difference between standing and lying view indicates the degree of muscular inefficiency (3) which prognoses a definite scope for correction through exercise treatment.

## Case presentation

In April this year, a 26-year-old male presented to the Dept. of Orthopedics with complaints of back pain caused by his Right Thoracic Idiopathic Scoliosis (Lenke 1) diagnosed one month prior. He had visited other spine clinics before coming to NIMS Hyderabad, and was suggested for surgery. Turned off by the invasive procedure, he was instead seeking alternative management and was referred to NIMS. The patient did not present any ligament laxity and did not undergo any genetic testing.

Family history disclosed consanguineous parents, but none of his siblings or cousins suffered from such a condition.

Whole spine standing x-ray AP and Lateral and traction films of the patient were evaluated using the digital spine software tool Surgimap.

## Presentation of pain

During the initial assessment, the patient reported pain and tenderness in the right upper mid back, bilateral shoulder girdle, and anterior joint. Prolonged sitting produced low back pain and subsequent walking was troublesome with an aching sensation in the right (upper anterior aspect) and left thigh (upper posterior aspect), hypertonic muscle, and taut band along the curve convexity noted on palpation.

Pain perception on a ten-point scale (VAS) was 8 on average.

## Musculoskeletal assessment

**Thoracic Convexity side (Right)—**Shoulder elevated, Posterior rotation of vertebral bodies palpable (Spinous processes at a higher altitude), Rib hump, Scapula protruded sitting on the rib hump and tilted anteriorly (Refer pre-intervention back view in supplementary digital material). Impingement and pain during arm elevation, unstable scapula during static and dynamic positions, Rotator cuff muscles felt inflamed and tender.

The Angle of Inclination was 15° at the thoracic curve apex (Scoliometer measurement).

**Thoracic Concavity side (Left)—**Posterior thorax appeared atrophied, anterior rotation of vertebral bodies palpable (palpating fingers at spinous processes felt a valley on this side), Shoulder depressed downwards, Rotator cuff felt tender and painful.

**Lumbar convexity side findings—**On inspection, the left buttock was more prominent.

Observation from side—left pelvis posterior and right pelvis anterior (Pelvic rotation in transverse plane).

On palpation, the left ASIS (Anterior superior Iliac spine) position was higher than the right ASIS, but the left PSIS (posterior superior Iliac spine) position was lower than the right counterpart (this proves Innominate rotation in the sagittal plane).

**Figure 1:**
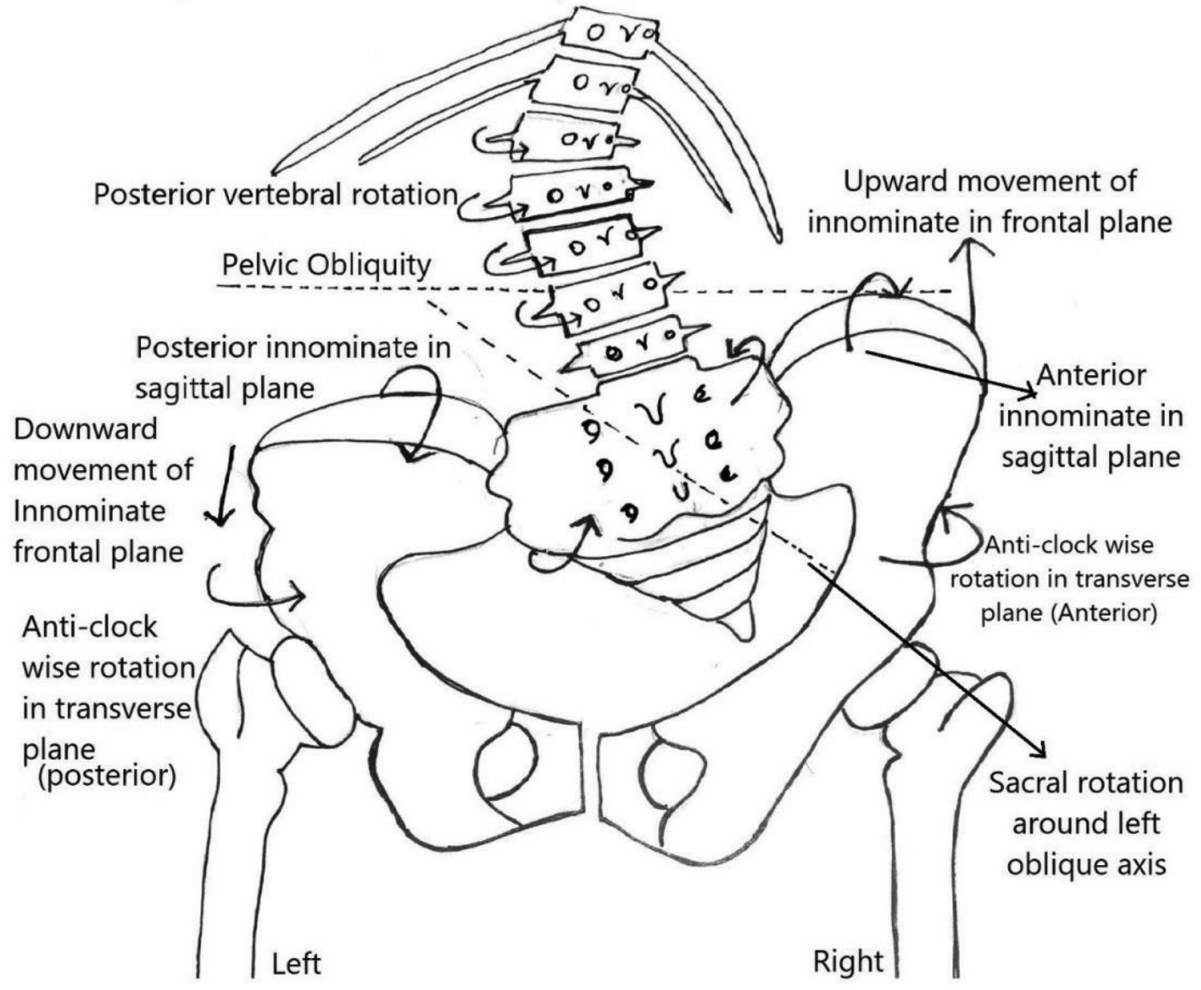
Directional Components of Pelvic obliquity as observed in a PA radiograph of left lumbar curve showing three dimensional motion of innominates and sacral rotation.

**Figure 2:**
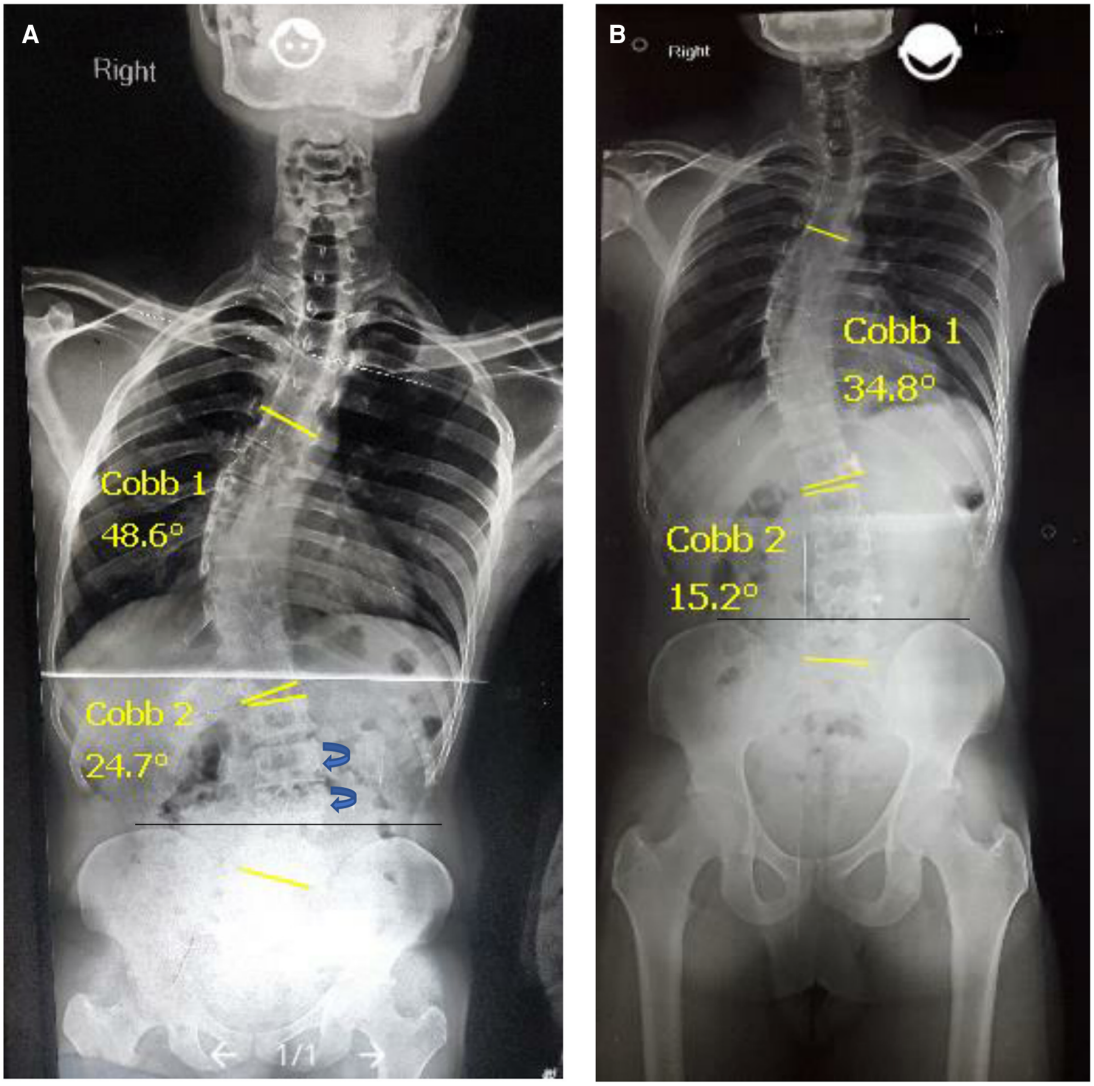
(**A**) Standing AP views, initial major cobb 48.6° showing pelvic obliquity. (**B**) Post-intervention major cobb 34.8° showing pelvic symmetry.

When the Innominate rotates anteriorly in the sagittal plane, the ASIS moves down and PSIS goes up, and vice versa. Here, the left Innominate rotated posteriorly and the right Innominate rotated anteriorly in the sagittal as well as transverse plane. This was visible in the pre-intervention radiograph as an asymmetrical width of both innominates, Figures 1, 2(A).

**Lumbar concavity side findings**—Waist indentation on right, Iliac crest moved up in the frontal plane to compensate for the left lumbar curve and maintain the coronal balance, making the right *Quadratus Lumborum muscle* tight.

### Limb length discrepancy

Standing Apparent limb length (*Xiphisternum* to *Medial Malleolus)*- Left > right.by 2 cm, Standing true length (*ASIS* to *Medial malleolus)*- Right > Left by 2 cm, Standing length (Greater *trochanter to medial malleolus*)- No discrepancy.

### Range of motion of thoracolumbar spine

Thoracolumbar flexion = 8 cm, Absence of extension range, Flexion = C7 to S1 Length in flexion—C7 to S1 Length in neutral standing, Extension = C7 to S1 Length in neutral standing—C7 to S1 Length in extension.

### Body mass Index

Body weight = 61 kg, Height = 172 cm, BMI = 20.6 kg/m^2^.

### Radiographic findings

Standing AP Major Cobb = 48.6°, Minor Left Lumbar Cobb = 24.7°, Traction Cobb = 29.8°, Degree of Flexibility = Standing Cobb ─Traction Cobb, i.e., 18.8° (Refer in supplementary digital material), Thoracic kyphosis = 9.9°, Lumbar lordosis = 54°.

Negative Coronal balance = (−20.1) mm on standing AP radiograph, C7 offset as a measure of Coronal alignment (Refer in supplementary digital material), Positive Sagittal balance = (15.7) mm on standing lateral radiograph, (SVA) Sagittal vertical axis as a measure of Sagittal alignment, measured as the horizontal distance between C7 plumb line and Central Sacral vertical Line (CSVL).

### Pulmonary function assessment

The predicted value of FEV1 = 4.01 liter, PEF = 568 L/m as per height, weight, gender, and age, (Measured on a smart one Spirometer).

Achieved Value (FEV1) = 2.68 liter (67%), PEF = 550 L/m.

## Methods

After the initial assessment, the patient signed a consent form to participate in the study and undergo exercise treatment. The treatment intervention consisted of institutional-based treatment of two hourly sessions once a week and a home program of the same protocol twice a day on other days of the week for 8 months. Around 12 sessions were spent releasing the myofascial knots that restored the optimum resting tone of the hypertonic longissimus thoracis, enabling pain-free performance and effective learning of the exercises.

The patient was encouraged to attain the position repeatedly as in *curve specific correction in sitting* with maximum effort for self-release and self-awareness of the corrected position (Refer image in supplementary digital material). After habituation and improved tolerance to exercises, the frequency of each exercise was slowly increased to 10 with an increasing hold period. Subsequent to myofascial release, breathing with core and aerobics was performed for warming up. On completion of all exercises, breathing with core was performed once more for stabilization of the newly attained range.

The exercise protocol consisted of the following techniques:

1. **Myofascial Release-**Para spinal muscles on the convex side of the curve, Upper trapezius of right side, Rotator cuff of both side, Rectus femoris of lumbar concave side, hamstring of lumbar convex side.

The above muscles were released using fingers, wrist pad, knuckles, elbows, and a manual massager.

2. **Breathing Exercises-**(a)Valsalva breathing (b) Breathing with core
  (a) **Valsalva Breathing**- strengthens the diaphragm isometrically.
  (b) **Breathing with core-**This is the most important technique to strengthen the deep, local intervertebral muscles like ***Rotators, Intertransversarii*, *and Multifidus***. In a recumbent position, the curve corrects to some extent, so the muscles are better aligned to develop optimal tension.

This is carried out in a supine knee bent position, with arms by the side, and elbows kept at 90°. The subject squeezes the bowel and bladder to engage the ***pelvic floor muscles*,** elevating them upward, and presses the navel down to recruit the ***Transversus Abdominis.*** Both the feet and elbows press down towards floor to facilitate the core at the lower abdomen, chest, and neck level. While engaging these core muscles, the patient tries to breathe in and out. This pulls the ***Diaphragm*** origin down and facilitates ***Diaphragmatic excursions*** against the abdominal and pelvic pressure. The wave of contraction of all major core muscles engages the local deep muscles of the whole spine. This is better performed on an empty stomach for at least 15 min.

3. **Aerobics:** (a) Yoga Cycling (b) Lunges to standing
  (a) **Yoga Cycling.**
  (b) **Lunges to standing**—Lumbar convex side leg forward, bringing the posteriorly driven innominate anterior and strengthening the quadriceps of this side, with the other leg backward in line with the body (5 min).
4. **Optimal Strengthening of Quadratus Lumborum**

Lying on the side with lumbar curve down, a towel roll kept underneath the curve to stretch the upper side Quadratus lumborum muscle, down leg flexed at hip and knee, upper leg lifted to a height just along the body line to strengthen the shortened muscle from an optimal position (Refer image in supplementary digital material).

5. **Stretching**

*Upper Trapezius*—Self -stretch of right-side using the Muscle Energy Technique of Post Isometric relaxation.

6. **Optimal Strengthening of Scapular Stabilizers**

The prone arm is lifted in an 120° abduction and the Scapula tilted posteriorly by manual support for optimal torque development.

Benefits- Prevents the anterior tilting of the Scapula and strengthens the stabilizers force couple.

7. **Rotation reversal Exercise for the Major thoracic Curve**

Subject sitting, thoracic convexity side arm kept against a wall with the shoulder at 90-degree abduction, elbow at 90-degree flexion, concavity side arm is also held in the same position, convex side arm pushes against the wall from the front side and concave side arm pushes to the back against therapist resistance. Simultaneous resisted contraction of horizontal arm adductor muscles from thoracic convex side facilitates anterior vertebral rotation and horizontal arm abductor from concave side facilitates posterior vertebral rotation that reverses the vertebral malrotation (Refer image in supplementary digital material).

8. **Planks** (Front plank, Reverse plank, and Side plank**)**

A side plank is held, keeping convexity side down, which stretches the upper concavity side muscles and strengthens the convexity side muscles.

9. **Strengthening of Trunk Flexors & Extensors-**Performed in a neutral trunk position or quadruped position, head just clearing off the bed to avoid hyperflexion and hyperextension. The mechanical impact of scoliotic spine hyperflexion and hyperextension is explained below.

During spinal flexion, vertebral bodies glide anteriorly over adjacent bodies (4), but concave side body halves already moved anteriorly, so further anterior movement pushes the convex side body halves more posteriorly, making the rib hump more conspicuous as in the *Adams forward bending test*. Similar asymmetrical posterior gliding during extension (4) also exaggerates the curve.

10. **Curve-Specific Corrective Exercises**

This correction is performed in three positions, namely lying, sitting, and standing, based on the principles of “Fryette's laws on spinal coupling” (5).

The 1^st^ law says that “side bending with spine in neutral, results in rotation to the contralateral side”, so the vertebral bodies rotate to the side of convexity and spinous processes are directed to the concave side.

The 3^rd^ law states that “when motion is introduced to a joint in one plane its mobility in other planes is reduced”.

### Correction in supine lying

The corrective exercises have to be curve-specific, and correction of all curves has to be done simultaneously to achieve optimal straightening.

**Figure 3:**
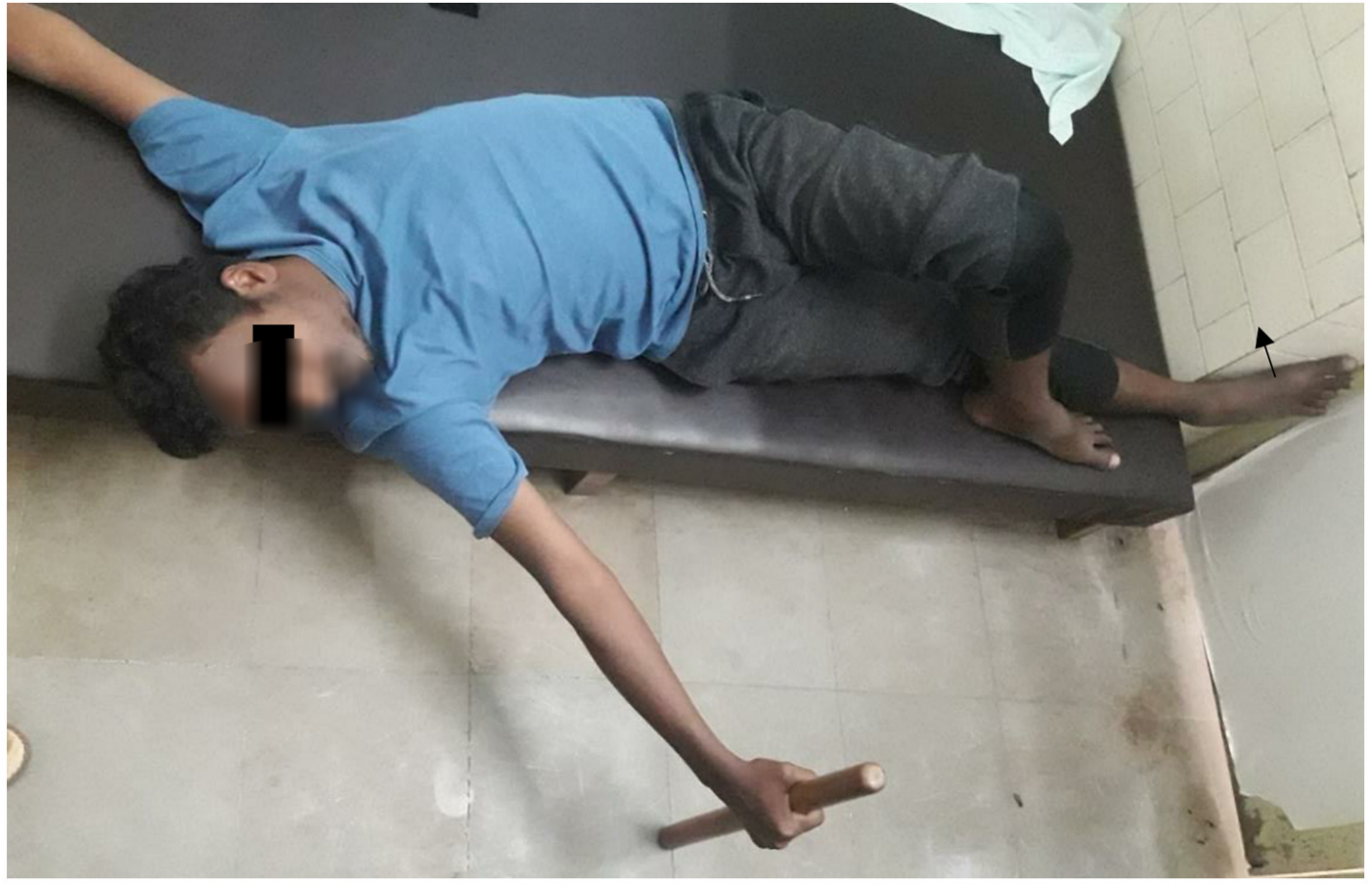
Curve specific correction in supine.

The recumbent position is a relatively corrected position with regard to muscle length-tension, and so is an optimal position to start corrective exercises (Figure 3).

#### Correction of pelvic obliquity

A radiograph is a prerequisite for this correction, and major thoracic curve correction has to be preceded by the correction of pelvic obliquity. Vertebral rotations in the compensatory lumbar curve are to the left posteriorly (Figures 1, 2), which drives the left part of the ring posterior (6). Transverse plane correction is the first correction to be achieved, which can beget the sagittal correction automatically. To achieve this, the posteriorly drawn left pelvis is brought forward by crossing the left leg over the right when lying down, flexed and adducted at hip and flexed at knee, left foot kept by the side of the right leg on the couch. The straight right leg with the crossed left leg over it now functions as a single lever arm with the pelvis corrected in the transverse plane.

#### Active correction of lumbar curve

Right foot pressure from the medial side against any fixed surface causes the lever arm to move at the *apex* of the curve, which acts as a *fulcrum* straightening the left lumbar curve by active facilitation of the lower lateral trunk flexors, drawing the right iliac crest down in the frontal plane, i.e., Frontal plane correction.

The active contraction of the lower lateral trunk flexors acts as a *counterpressure* during major thoracic correction. Meanwhile, transverse plane correction of the pelvic obliquity pulls the lower lumbar vertebral bodies anterior in transverse plane, thereby obeying Fryette's 3rd law, and are stabilized to move in another plane. Both factors prevent any possibility of exaggeration of a lower curve while the subject bends to the thoracic convexity side for thoracic curve correction. The same rule is applied to correction in all three positions.

#### Major thoracic curve correction

To reverse the upper compensatory vertebral rotations in the cervicothoracic region, the head is rotated to the right side, the left arm is elevated, and the major thoracic curve is straightened by bending to the right side in a supine position, performing the Muscle Energy Technique (MET) of convex side muscles against the subject's own arm resistance generated by pushing a small stick stood on the ground or pushing against any fixed surface (5). The patient performs isometric contractions of the right erector spinae muscle group at the limit of lateral bending for a count of 10 (MET), with Valsalva breathing. Valsalva is performed by holding the breath during the contraction. The Diaphragm undergoes an isometric contraction during the procedure and releases Diaphragmatic adhesions and strengthens the *convex side spinalis thoracis.* After around 10 sessions, the patient performs side bending with normal breathing.

### Correction in sitting

**Figure 4:**
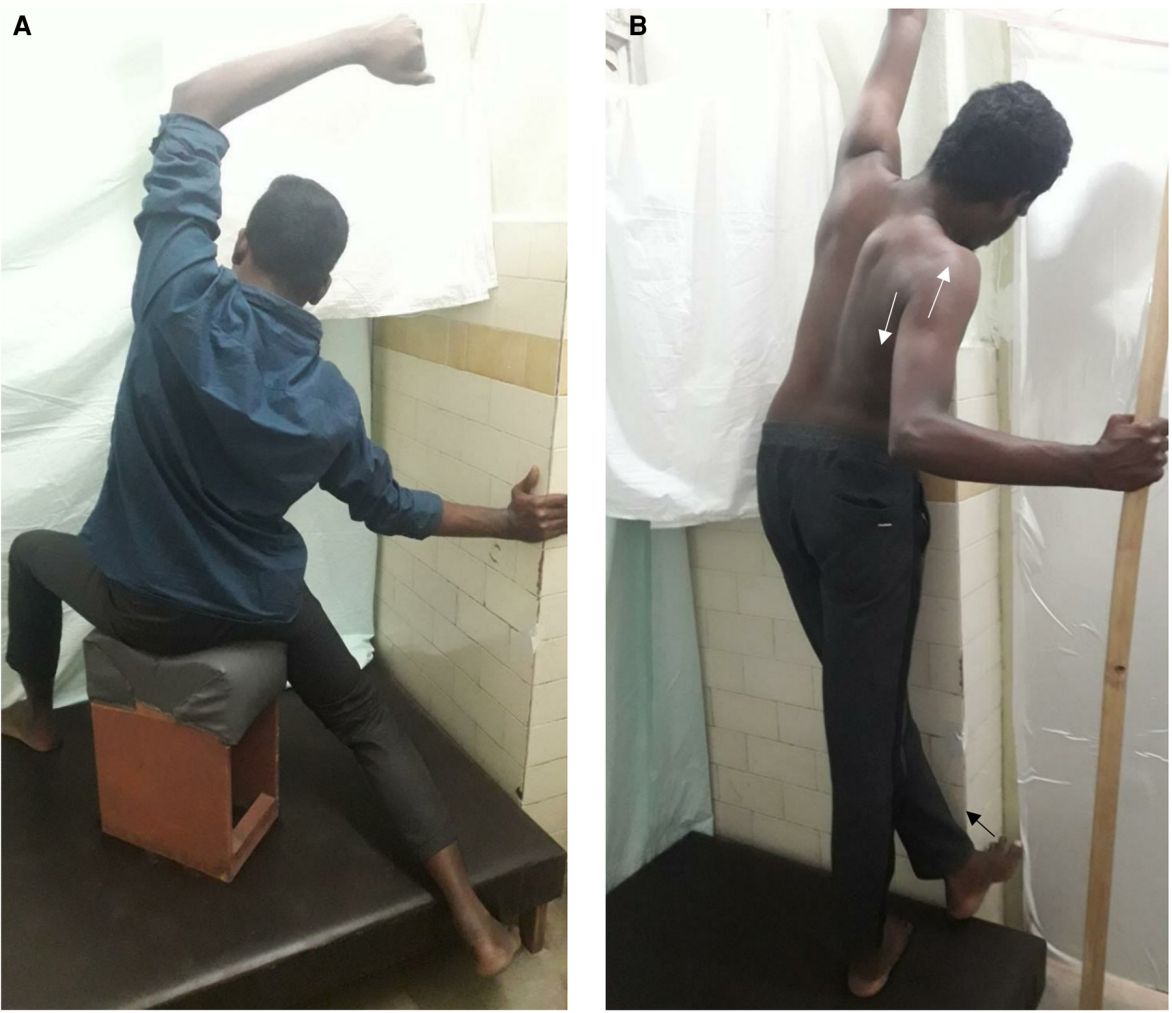
(**A**) curve specific correction in sitting. (**B**) Curve specific correction in standing.

Corrective exercises in sitting are useful as the subject can sit in a corrective position in both his home and workplace. Bending ipsilaterally when sitting is a gravity-aided movement, and so is helpful for controlling creep on the convex side muscles (Figure 4A).

#### Pelvic and lumbar correction

The patient sits on a small stool or arm-less chair, with the left leg kept in the front at the edge, and right leg kept as far back as possible so that the left side pelvis and lumbar bodies automatically come forward. The left foot is used to press any fixed surface from the lateral side to create active pressure for curve straightening. This sitting posture prevents back pain and helps prolonged sitting in a desk job.

#### Thoracic curve correction when sitting

The patient bends to the convex side while the right trunk is kept forward and left trunk is held backward, performed similarly as when lying.

### Correction in standing

**Lumbar and Pelvic correction*—***Subject stands on the right leg, kept back and adducted. The left foot taken forward pushes against a wall or any fixed surface from the lateral border (Figure 4B).

**Major Thoracic and upper Cervicothoracic Correction-**Same as in the sitting position.

Dosage of Exercises- Repetitions and hold period progressed as pain reduced.

### Ergonomic positions to be maintained during Activities of Daily Living

**Lying on left side:** Lying with left compensatory lumbar curve down, a rolled towel underneath the body as in optimal strengthening of the Quadratus lumborum, left arm kept elevated along the body line. This position stretches the thoracic curve on the concave side and quadratus lumborum on the right side.

**Lying on Right side:** Lying with convex thoracic side down, down arm forward, upper arm back, upper leg forward, and down leg backward. This position facilitates correct rotation from the thoracic and lumbar regions.

**Sitting and Standing**: The lumbar convex side leg is kept forward as in corrective exercises in sitting and the same side leg is kept against the tabletop, bent at the knee, to keep it at a higher level than the other knee. This position corrects the pelvic obliquity in three dimensions and helps sustain prolonged pain-free sitting.

During standing, maintaining a walk standing position keeping lumbar convex side leg forward and the other side leg backward corrects the pelvic obliquity.

### Carrying weights and picking objects up:

Weights are hung from the thoracic convex side hand to shift the line of gravity (LOG) from the concave side towards the center, reducing the gravitational torque, while picking up objects, bringing the Right trunk to the front.

**Staircase walking**-Lumbar convex side leg to progress first.

## Results

### Pain perception

Post intervention assessment revealed significant relief from all types of pain; the subjective VAS rating reduced from 8 to 1 and the patient could hold a new desk job which he had previously quit because of pain.

The patient stated, “I am not getting any types of pain, and not worried about my future now as I know how to control this condition on my own”.

### Postural and musculoskeletal changes

There was a less protruded Scapula on the Right, left Scapula pronounced, and Symmetrical pelvic position (Correction of pelvic obliquity apparent from symmetrical, equal-sized innominates in post-intervention x-Ray), Figure 2(B).

Angle of Inclination reduced to 10°.

Correction of Apparent and True Limb length discrepancy by 1.5 cm.

Thoracolumbar flexion and extension range enhancement by 3.5 and 3 cm respectively.

Body weight reduced by 1 Kg with an improved body composition.

### Curve correction

Significant reduction of major Cobb angle by 13.8° from 48.6° to 34.8°, Minor Cobb by 9.5° from 24.7° to 15.2°.

Thoracic Hypo Kyphosis of 9.9° normalized to 37.8°. Lumbar Lordosis of 54° improved to 69°.

### Regaining of global spinal balance

Negative Coronal balance of (−20.1) mm reduced to −2.7 mm.

Positive Sagittal balance of 15.7 mm reduced to 11.5 mm.

FEV1 of 2.68 liters (67%) changed to 3.00 liters (72%). PEF improved by 18 liters/min.

## Discussion

Spinal pain syndrome in scoliosis can originate from different anatomical sites, such as annular and facet joint stress, neural tension, and fibrotic muscle, and physical therapy as a form of treatment is recommended by SOSORT and SRS to control this (1, 6).

The varied regional pain of the present case can be explained on the basis of literature and clinical findings.

The upper mid back pain is assumed to originate mainly from the fibrotic muscle along the convex side of the curve that got overburdened due to persistent work in the frontal plane to counteract the gravitational torque shift on the concave side. Stretch weakness also reduces the optimal tension producing abilities turning it hypertonic with chronic inflammation and fibrosis restricting motion and correction.

An elevated shoulder and rib hump changes the orientation of the lower trapezius and Serratus anterior force couple, making the scapula unstable. Girdle and Rotator cuff pain could be due to a tight upper trapezius and overactive cuff muscle to stabilize the scapula, creating inflammatory changes.

Shoulder depression on the thoracic concavity side submits the capsule and cuff to increased gravitational traction, forcing the cuff muscle to contract maximally to stabilize the head in the socket, making them inflamed and painful (6).

The lower limb pain could arise from altered muscle length and stressed ligaments and capsules attributed to tri- plane pelvic distortion. Sagittal plane *distortion* (Left Innominate posteriorly, Right Innominate anteriorly) increases tension in the *right Hamstring*, *right Sacro tuberous ligament*, and left *Rectus Femoris muscle* and decreases tension in the *left hamstring*, *right Rectus Femoris* muscle, and *Iliotibial band* (6), increasing energy expenditure and creating inflammatory changes.

Transverse and Frontal plane pelvic obliquity alters the bony landmarks from which true length is measured, and so creates a false picture of true limb length discrepancy.

All components of muscular failure with spinal pain were effectively handled by the myofascial release, stretching, strengthening exercises, and proper ergonomics facilitating postural re-education.

Reduction of low back pain could be attributed to lumbar curve straightening which depreciated L3–L4 end plate obliquity angles (7).

Literature studies confirm the efficacy of exercises in halting the progression rate of idiopathic scoliosis in puberty and in adulthood, but reduction of the Cobb angle around the end of the growth period and during adulthood achieved after a long treatment duration is often within the range of measurement error, i.e., <5° (8, 9). However, in a case study, Negrini et al. showed documented evidence of curve reduction of 18° by scoliosis-specific exercises in an adult (10).

Straightening of a curve by 13.8° in the major curve and 9.5° in the minor curve of this adult case after 8 months is not a deviation from the previous findings but a strengthening of treatment efficacy.

The curve correction could be due to simultaneous stretching of concave side soft tissue structures at both the thoracic and lumbar curve (the degree of flexibility = 18.8°) and stabilization of the attained range by strengthening of smaller, deeper local muscles and larger, superficial, global antigravity muscle.

The superficial hypertonic longissimus thoracis inhibits the deeper local stabilizers that help brace the spine internally (11). Subsequent to myofascial release, this deeper system slowly improved its bracing action by “breathing with core” developing endurance.

Improvement in other morphological parameters, like posture and aesthetics, and functional parameters like ROM, flexibility, body mass composition, pulmonary function measures, normalization of thoracic kyphosis, and sagittal and coronal balance (12) are advantages of curve correction. The treatment also provided pain-free activity which improved global balance, function, shock-absorbing mechanics, and possibly further pain control.

The limitation of the treatment method is that the exercises cannot be performed without institution-based therapy sessions in the beginning, as they are needed to release myofascial adhesions and effectively learn the corrective techniques. Once the patient masters self -release with self- awareness and breathing techniques with all corrective exercises, they can be performed anywhere with continued improvement over time.

The limitation of the case is that this improvement is shown in only a single case.

## Conclusion

This case report proves the plausibility of curve reduction in adult cases with a biomechanically designed exercise protocol. The protocol can correct the multiple signs and symptoms of idiopathic scoliosis, reverse all dimensions of deformity, control spinal pain syndrome of scoliosis, and provide a sustainable correction. Irrespective of variable etiological factors behind the initiation of Idiopathic Scoliosis, the musculoskeletal dysfunction sequelae are similar, and they can be treated against this background. The corrective exercises are comprehensive, curve-specific, and feasible to be applied for all Lenke curve types by changing the direction to a suitable position as per the needs of the patient.

More time is required to assess the level of improvement beyond a 1-year period of regular adherence to the protocol.

## Data Availability

The original contributions presented in the study are included in the article/Supplementary Material, further inquiries can be directed to the corresponding author/s.
